# Stress Related to COVID-19 and Loneliness Among Middle-Aged and Older Adults in the United States

**DOI:** 10.1093/geroni/igab046.1140

**Published:** 2021-12-17

**Authors:** Sukyung Yoon, Neely Mahapatra

**Affiliations:** University of Wyoming, Laramie, Wyoming, United States

## Abstract

Intensified levels of stress and loneliness have been attributed to the COVID-19 pandemic (Havnen et al., 2020; Luchetti et al., 2020). Moreover, loneliness has been reported to exacerbate psychological and physical health issues (Holt-Lunstad et al., 2015). The current research aims to investigate the impact of stress-related to COVID-19 on loneliness. The roles of age, sex, living arrangements, health, and resilience were also investigated. Data was collected on 267 middle-aged and older adults (ages 45 through 88) living in the U.S during COVID-19. A path analysis was employed. For both the direct and indirect effects, 95% confidence intervals were estimated using bootstrapping (a bootstrap sample of 1,000 was specified). Model fit was acceptable. X2 (5) = 7.913, p > 0.05, CFI=0.972, RMSEA =0.047. Regarding direct effects, the results indicate that COVID-19 related stress (hereafter stress) was negatively associated with perceived good health (hereafter health) (β = -.213, p<0.001). It was also found that health was positively associated with resilience (β = .324, p<0.001). Being male was positively associated with resilience (β= .144, p<0.05), and resilience was negatively associated with loneliness (β= .230, p<0.001). Meanwhile, stress had negative indirect effects on resilience, whereas stress had positive indirect effects on loneliness. Finally, health and being male had negative indirect effects on loneliness. The findings indicate that health practitioners and service providers should develop programs to improve and maintain good health, resilience, and social support among middle-aged and older adults during the COVID-19 pandemic. Moreover, gender-based services are also needed.

